# Predictors of Poor Outcome among Critically Ill COVID-19 Patients: A Nationally Representative Sample of the Saudi Arabian Population

**DOI:** 10.3390/jcm11102818

**Published:** 2022-05-17

**Authors:** Masaad Saeed Almutairi, Ahmed M. Assiri, Omar A. Almohammed

**Affiliations:** 1 Department of Pharmacy Practice, College of Pharmacy, Qassim University, Qassim 51452, Saudi Arabia; mas.almutairi@qu.edu.sa; 2 Health Volunteering Center, Ministry of Health, Riyadh 11176, Saudi Arabia; aasssiri@moh.gov.sa; 3 Department of Clinical Pharmacy, College of Pharmacy, King Saud University, Riyadh 11451, Saudi Arabia; 4 Pharmacoeconomics Research Unit, College of Pharmacy, King Saud University, Riyadh 11451, Saudi Arabia

**Keywords:** COVID-19, ICU, poor prognosis, mortality, risk factors

## Abstract

The outbreak and continuing impact of COVID-19 have significantly increased the rates of hospitalization and admissions to intensive care units (ICU). This study evaluates clinical outcomes in critically ill patients and investigates variables tied to poor prognosis. A secondary database analysis was conducted to investigate the predictors of poor outcome among critically ill COVID-19 patients in Saudi Arabia. Multivariable logistic regression analysis was used to assess the association between various demographic characteristics, comorbidities, and COVID-19 symptoms and patients’ poor prognosis, as a composite outcome. A total of 2257 critically ill patients were identified (male (71.8%), and elderly (37.3%)). The mortality rate was 50.0%, and the composite poor outcome was 68.4%. The predictors of poor outcome were being elderly (OR = 4.79, 95%CI 3.19–7.18), obesity (OR = 1.43, 95%CI 1.1–1.87), having a severe or critical case at admission (OR = 6.46, 95%CI 2.34–17.8; OR = 22.3, 95%CI 11.0–45, respectively), and some signs and symptoms of COVID-19 such as shortness of breath, feeling fatigued or headache, respiratory rate ≥ 30/min, PaO_2_/FiO_2_ ratio < 300, and altered consciousness. In conclusion, identifying high-risk populations that are expected to have a poor prognosis based on their criteria upon admission helps policymakers and practitioners better triage patients when faced with limited healthcare resources.

## 1. Introduction

The current coronavirus disease (COVID-19) pandemic has resulted in high rates of hospitalizations and intensive care unit (ICU) admissions [1]. The disease is also associated with high mortality rates among critically ill patients worldwide [2]. Critically ill patients with COVID-19 have exhibited numerous complications, such as acute kidney injury (AKI), multiorgan dysfunction, and acute respiratory distress syndrome (ARDS); moreover, many of these patients have required mechanical ventilation [3,4,5]. Currently, the global number of COVID-19 patients is rising again, driving a critical need to increase the capacity of ICU beds. Accordingly, health organizations in many nations have increased the number of ICU beds to meet this need [6]; in Saudi Arabia (SA) in particular, the number increased by 43% in the capital city of Riyadh alone, mirroring significant increases around the country [7].

The severity of the disease varies among patients due to differences in the response of their immune system, existing comorbidities, and other factors [8]. Prediction of a poor outcome in patients infected with COVID-19, especially in the case of critically ill patients, is vital to provide the best possible patient care while managing the burden on healthcare systems. Globally, several studies have investigated predictors of poor outcomes for COVID-19 patients. For example, a single-center study in China found that being an age of over 65 years, smoking, critical disease status, and diabetes mellitus (DM) were associated with unfavorable outcomes in critically ill patients with COVID-19 [9]. A prospective single-center study conducted in New York city and Long Island concluded that male patients and those of older age, and those who have impairments in oxygen level at admission along with other inflammatory biomarkers were at high risk of having critical illness [10]. A multicenter study in the US showed that the male gender was associated with poorer outcomes in critically ill patients than the female gender [11]. A meta-analysis investigating predictors of poor prognosis among COVID-19 patients in a mostly Chinese population found that several biomarkers (C-reactive protein, interleukin-6, and D-dimer) and comorbidities (such as hypertension, diabetes mellitus, and chronic obstructive pulmonary diseases) were associated with poor patient prognosis [12]. Another large meta-analysis assessed the association of prognostic factors with poor patient prognosis and found that the following factors were correlated with at least one poor outcome: angiotensin-converting enzyme inhibitors, obstructive sleep apnea, pharyngalgia, history of venous thromboembolism, gender, coronary heart disease, cancer, chronic liver disease, chronic obstructive pulmonary diseases, dementia, any immunosuppressive medication, peripheral arterial disease, and rheumatological disease [13]. Moreover, data from studies conducted in different countries were combined in two recent meta-analyses whose authors reported that some demographic data (i.e., male gender and older age), hypertension, diabetes mellitus, cardiovascular diseases, and malignancy were associated with poor outcomes [14,15].

Several studies set in SA have described the clinical characteristics of COVID-19 patients, though most of them were limited by small sample size [16,17]. The overall mortality rate among critically ill patients in SA was estimated to range between 25% and 42% and was higher among patients on mechanical ventilation [18,19]. Although understanding the significant risk factors that could help predict which patients are at risk of poor outcomes in different populations is important, limited studies are available from SA evaluating such predictors in critically ill patients. A single-center retrospective study that included critically ill patients found that the odds of mortality were higher in older patients [20]. Another single-center study found higher odds of mortality in patients having advanced age, end-stage renal disease, and low oxygen saturation upon admission [21]. Moreover, a retrospective study in which 27.5% of the participants were critically ill patients assessed predictors of poor outcomes and found that older age, the existence of comorbid conditions, and oxygen saturation below 94% upon admission were associated with poor outcomes [22]. Thus, the current study was conducted to investigate the clinical outcomes of critically ill patients with COVID-19 in SA and assess risk factors for poor outcomes.

## 2. Materials and Methods

### 2.1. Study Design and Setting

A secondary database analysis was conducted to investigate the clinical outcomes of critically ill patients with COVID-19 and assess the risk factors for poor outcomes. The data were retrieved from the Saudi Ministry of Health database for hospitalized patients with COVID-19 who were confirmed with real-time reverse-transcription polymerase chain reaction (RT-PCR) test. The Central Institutional Review Board at the Saudi Ministry of Health reviewed and approved the study protocol (IRB Log No: 21-96 M).

### 2.2. Subjects, Database, and Outcome

All patients in the database who had been hospitalized between March 2020 and January 2021 were screened for inclusion. Data for adult (≥18 years) and critically ill patients were included in the analysis. However, data related to pediatric patients and pregnant women were excluded from the analysis. Critically ill patients were defined as all patients who were admitted to ICU during their hospitalization. The database included information on patients’ demographics, ICU admission, risk factors, comorbid conditions, and the severity stage of the COVID-19 case at the time of admission, as well as the therapeutic intervention and oxygen-supplementation therapy received during hospitalization. Various complications that affected these hospitalized patients, such as septic shock, AKI, ARDS, multiorgan failure, and the patients’ status at discharge (survived or deceased), were also recorded. The study defined a poor outcome as a composite outcome, either having at least one of these inpatient complications (AKI, ARDS, multiorgan failure) or dying before discharge.

Upon admission to hospital, patients with COVID-19 were classified into three stages of severity based on their existing COVID-19 symptoms at admission. Stage A: mild-to-moderate symptoms only (high temperature, a new or continuous cough, a feeling of being breathless or short of breath, a loss of the sense of smell, and feeling tired, having muscle aches or a headache); Stage B: severe symptoms (respiratory rate ≥ 30/min for adults, blood-oxygen saturation ≤ 93%, PaO_2_/FiO_2_ ratio < 300, and lung infiltrates > 50% of the lung field within 24–48 h of presentation); and Stage C: critical symptoms (ARDS, sepsis, multiorgan failure, and altered consciousness). Milder symptoms were omitted from documentation during admission if patient presented to the hospital with severe or critical symptoms (e.g., if a patient presented with symptoms suggesting ARDS, cough, or fever were not recorded as symptoms).

### 2.3. Statistical Analysis

Descriptive statistics were used, including mean (±SD) for continuous variables, such as age and body mass index (BMI), as well as frequencies (%) for other patient characteristics and outcomes. Multivariable logistic regression analysis was used to investigate the association between the patient or disease characteristics (demographics, risk factors, COVID-19 symptoms, etc.) and the composite poor outcome (dependent variable). All statistical analyses were performed at a significance level of α < 0.05, and the results from the regression were presented as odds ratios (OR) with a 95% confidence interval (95%CI). The data were managed using Microsoft Excel, version 2010 (Microsoft Corp., Redmond, WA, USA), and all statistical analyses were performed using the SAS^®^ software, version 9.4 (SAS Institute Inc., Cary, NC, USA).

## 3. Results

### 3.1. Patient Characteristics and Risk Factors

Out of 4125 hospitalized patients with COVID-19 in the database, 2257 critically ill patients were included in the analysis. The majority (71.8%) of included patients were male. The average age of these patients was 59.7 (±14.6) years, and more than one-third of them were elderly (≥65 years). The mean BMI was 29.0 (±6.4), while 17.5% of the included patients were morbidly obese (BMI ≥ 30). The most common comorbidities were diabetes mellitus (63.85%) and hypertension (57.95%). Almost half of the patients (46.3%) presented to hospitals with Stage B (severe) symptoms; the rest of the patients presented with either Stage A (mild-to-moderate) or Stage C (critical) symptoms. Table 1 displays the patients’ characteristics and risk factors.

### 3.2. Therapeutic Interventions and Oxygen-Supplementation Therapy

Dexamethasone (80.0%) and favipiravir (70.1%) were the most commonly used medications in these patients. Since all patients in this study were critically ill, almost all of them (97.6%) needed oxygen-supplementation therapy. The most frequently used oxygen therapy was nasal or face mask (69.3%) followed by invasive mechanical ventilation (57.5%) and high-flow face mask (56.9%). The administered therapeutic interventions and oxygen therapy are depicted in Table 2.

### 3.3. Patients’ Outcomes

Among the included critically ill patients, ARDS was the most common complication, affecting 1399 (62.0%) of these patients, followed by septic shock 855 (37.9%). AKI and multi-organ failure affected 25.4% and 22.0% of these patients, respectively. In addition, 1130 patients, representing one-half of these critically ill patients, died in the hospital. Overall, the composite poor outcome occurred in 1543 patients, representing more than two-thirds of these patients (68.4%). The mean duration of hospitalization for these patients was 16.4 (±12.8) days and the length of stay in the ICU was 11.4 (±11.4) days.

### 3.4. Predictors of Patients’ Composite Outcome

Older patients had higher odds of having poor outcomes, and patients aged over 65 years had more than four times the odds of having the composite outcome compared to patients aged 40 years or younger (OR = 4.79, 95%CI 3.19–7.18). In addition, obesity was associated with poor outcome (OR = 1.43, 95%CI 1.10–1.87). Furthermore, the results indicate that patients who were in Stage B or C on admission had more than six times the odds of exhibiting a composite outcome compared to those in Stage A (OR = 6.46, 95%CI 2.34–17.80; OR = 22.3, 95%CI 11.0–45.3, respectively). On top of that, several signs and symptoms such as shortness of breath (OR = 1.80, 95%CI 1.00–3.22), feeling fatigue or headache (OR = 2.16, 95%CI 1.47–3.17), respiratory rate (RR) ≥30/min (OR = 1.42, 95%CI 1.02–1.96), PaO2/FiO2 ratio <300 (OR = 1.65, 95%CI 1.21–2.26), and altered consciousness (OR = 2.29, 95%CI 1.30–4.01) were predictors of poor outcome. Although having sepsis at admission was significantly higher among patients with poor composite outcomes compared to patients without (85.7% vs. 14.3%; *p* < 0.001; OR = 3.12, 95%CI 2.22–4.38), it was not a positive predictor for poor composite outcome in the multivariable analysis. The distribution of patients with composite outcome based on their characteristics along with the ORs from the multivariable logistic regression is summarized in Table 3. The sensitivity and specificity, positive and negative predictive values, and the ROC value and for the multivariable logistic regression model were provided in the Supplementary Materials.

### 3.5. Composite Outcome Stratified by Gender and Stage at Admission

Overall, the composite outcome occurred in 1543 (68.4%) patients of the critically ill patients in the study, of whom only 28.2% were females (*p* = 0.74). No difference was observed between male and female in the incidence of composite outcomes from the stratified analysis nor the multivariable analysis. Patients who were admitted to hospital with severe (Stage B) or critical symptoms (Stage C) were significantly (*p* < 0.001) more likely to have poor composite outcomes (Table 4).

## 4. Discussion

The study evaluated the clinical outcomes in critically ill patients from SA and investigated variables predicting these poor prognoses. Older age, obesity, being identified as a severe or critical case upon admission, and some signs and symptoms on admission (shortness of breath, feeling fatigue or headache, RR ≥ 30/min, PaO_2_/FiO_2_ ratio < 300, and altered consciousness) were associated with poor outcomes in these critically ill patients. These findings are comparable to those from previous studies [9,14,15,20,21]. Although diabetes mellitus and hypertension were the most often-seen comorbid condition in the study, affecting more than half of these patients, they were not significant predictors of poor outcome in this population in the multivariable logistic regression. This outcome was inconsistent with reports from other studies that found diabetes mellitus to be a significant predictor for an unfavorable outcome in COVID-19 patients [9,15].

The overall mortality rate in the study was 50%; notably, the study included only critically ill patients who were admitted to the ICU. This number was higher than the mortality rate of around 42% reported by Al Suliman et al. in a local study that included 560 critically ill patients [18]. However, similar or even higher mortality rates, ranging between 53% and 67%, were reported in studies from Asia, Europe, and the US for critically ill patients with COVID-19 [3,23,24]. In contrast to Al Sulaiman et al.’s study, which examined data for patients hospitalized in only two large medical centers, this study included data from 29 hospitals around the country.

In these critically ill patients with COVID-19, older patients had a higher risk of having a poor outcome compared to patients aged younger than 40 years. This result was in accordance with findings from both international [25,26] and local studies [27]. Moreover, this study found that even younger patients (41–64 years) had a higher risk of the composite outcome compared to patients younger than 40 years. Although several reports have shown that male gender was associated with higher odds of poor outcomes compared to female [28,29], our analysis did not find this result among critically ill patients. In accordance with our finding, a retrospective study including ICU patients from China did not find a difference in the incidence of poor outcomes between males and females [30]. While no difference was observed between males and females in the incidence of composite outcomes, neither in the stratified analysis nor the multivariable analysis, it is worth noting that more males were included in the study, which indicates a higher risk of admission to ICU among males compared to females.

Several studies reported that critically ill patients with COVID-19 were at risk of having various complications, such as ARDS, AKI, acute liver failure, acute cardiac injury, septic shock, or secondary infections [11,31,32,33]. In the current study, the most common complications were ARDS and septic shock, followed by AKI. Owing to our population of critically ill patients with COVID-19, we expected to report a high percentage of ARDS. The findings of a high percentage of ARDS or respiratory failure in this study were consistent with those of another study that reported that the incidence of ARDS, which require oxygen supplementations, was the most concerning complication among critically ill patients with COVID-19 [34]. Although mechanical ventilation is considered a lifesaving intervention for patients with critical COVID-19 cases [26], several studies emphasize that the need for mechanical ventilation is an indicator of poor outcome [35,36,37]. Since most of our critically ill patients had ARDS (62%) and most of these patients needed mechanical oxygen therapy (57.5% received IMV) to sustain their life, we came to a different logical interpretation for the association between mechanical ventilation and the incidence of poor outcomes. Thinking of the condition (COVID-19 and the risk of developing ARDS), the intervention (mechanical ventilation), and the need for and time to start using this intervention (critically ill patients with low oxygen level), the use of mechanical ventilation among critically ill patients with COVID-19 will always be associated with poor outcomes. However, it is not because it has no important role or added value in these patients; rather, we believe it is needed and has an important role in therapy, but since it is used in patients with ARDS and these patients usually have poor outcomes, this will make it always look like an unbeneficial or even risky intervention when we assess the association between its use and the incidence of poor outcomes.

About one-quarter of these critically ill patients developed AKI during their hospitalization, making it the third most common complication in this population. In comparison to our results, Al Sulaiman et al. found that 46% of their patients had AKI [18]. Similarly, a large multicenter study in the US with 4221 critically ill patients found that AKI occurred in 56% of their patients [38]. Several studies have proposed the mechanisms of AKI development in COVID-19 patients, which include a higher level of soluble tumor necrosis factor, stimulation of inflammatory mediators and cytokine storms, which can cause a microvascular injury and AKI; moreover, the virus can target angiotensin-converting enzyme 2, which is found in the kidney, and can even directly infect the kidney [39,40]. Although our finding was not close to previous estimates, COVID-19 patients are clearly at high risk of developing AKI. Thus, these patients need close monitoring of their renal function during hospitalization to timely diagnose and manage these cases of AKI and avoid long-term complications.

The current study stratified patients based on their COVID-19 symptoms at admission to provide practitioners with guidance concerning the need for the appropriate level of care [41]. Although this stratification method is unique to our system and was not built to predict patients’ outcomes, the idea of risk stratification based on patients’ factors has been used in a previous study in which the researchers constructed two risk-stratification score systems, STPCAL and TRPNCLP, to predict COVID-19 patients’ duration of hospitalization and progression [42]. However, using two methods is much more complicated than using one simple three-stage classification system based on existing COVID-19 symptoms. In our study, patients admitted to hospital with critical symptoms (Stage C) had a much higher odds of having poor composite outcomes than patients admitted with mild-to-moderate (Stage A) or severe (Stage B) symptoms. However, this was not the main objective of the study; thus, further investigation and evaluation of this stratification system is needed.

It is worth noting that variations in clinical outcomes among patients with COVID-19 were attributed to several factors, including the genetic variants of SARS-CoV-2 [43]. The risk of disease severity and mortality were higher in variant of concern compared to non-variant of concern, including the wild type (Wuhan) [44]. Several studies evaluated the effect of different variants on clinical outcome and transmissibility of the virus, and some variants were more dominant than others. Compared to the wild type, the Beta, Alpha, Delta, Gamma, and Omicron variants had higher transmissibility and were associated with worse clinical outcomes [43,45,46,47,48]. Although no data on the type of variants were available for our sample, we believe that the wild type was the most common variant in our sample. Therefore, future analysis of the impact of different SARS-CoV-2 variants on clinical outcomes in Saudi Arabia is warranted.

In summary, this study identified the predictors of poor outcomes in critically ill patients with COVID-19, providing essential information for policymakers and practitioners that will support the creation or updating of clinical guidelines or protocols to better determine patients’ level of severity. Although this study represents one of the largest or the largest investigation concerning critically ill COVID-19 patients in SA and the region, it has some limitations that must be underlined. For example, the study’s utilization of a database offering data collected retrospectively to ensure clinical excellence and assess patients’ outcomes limits the validity of these findings based on the quality of the data collected. The laboratory and diagnostic data for these patients were not available in the database, prohibiting the validation of some of the findings in the study, such as the incidence of AKI, which characterizes one of the limitations for studies using secondary databases. In addition, there were no data about the causative variant of the virus which limited the analysis and interpretation of the results; the existence of this data would have allowed better understanding of the variation among variants on critically ill patients’ outcomes. In addition, no data were collected to identify the existence of chronic kidney diseases before admission which may affect the estimate for the occurrence of AKI in the study. Although the incidence of sepsis was associated with higher odds of poor outcomes in the univariate analysis which we expect and understand, its effect in the multivariable analysis was in the opposite direction, which has no clear justification in our opinion. Dropping sepsis, as a variable, from the multivariable model did not make a difference in the significance of the model (it remained significant) nor the ROC (0.747 vs. 0.745), which means sepsis is not as important as other variables in the model. However, we preferred to keep it in the model for the sake of completeness and emphasize the need for further investigation to explain this unexpected result. Because pediatric and pregnant patients were excluded from the study, the study findings should not be generalized to these special populations, and further study is needed for these patients. Lastly, the results might be generalized with caution to the vaccinated population because the analyzed data were collected before the mass vaccination of adults in SA. Thus, it is not known if the results would be the same if some or all of these critically ill patients were vaccinated before their infection.

## 5. Conclusions

The study identified several possible factors that were associated with poor outcomes in critically ill patients with COVID-19 in SA. The use of severity scale might be helpful in identifying patients at risk of poor outcomes but it needs further evalaution and validation. The recognition of high-risk populations through an evaluation of different disease variables and patients’ characteristics that are associated with poor outcomes may help policymakers and practitioners better triage patients during this pandemic, especially in the context of limited healthcare resources.

## Figures and Tables

**Table 1:** Patients’ characteristics (*n* = 2257).

Characteristic	Number (%)
**Age, mean (SD)**	59.7 (14.6)
≤40 years	218 (9.7)
41–64 years	1202 (53.3)
65–75 years	462 (20.4)
≥75 years	375 (16.6)
**Body mass index, mean (SD)** [*n* = 1683]	29.0 (6.4)
BMI < 25	452 (6.9)
25 ≤ 30	669 (39.8)
30 ≤ 40	474 (28.2)
≥40	88 (5.1)
**Gender**	
Male	1621 (71.8)
Female	636 (28.2)
**Nationality**	
Saudi	868 (38.2)
Non-Saudi	1394 (61.8)
**Risk factors**	
Diabetes mellitus	1441 (63.9)
Hypertension	1308 (58.0)
Elderly (aged 65 years or more)	841 (37.3)
History of cardiovascular disease	521 (23.1)
Obesity or severe obesity	395 (17.5)
History of pulmonary disease	343 (15.2)
Underlying end organ dysfunction	281 (12.5)
Immunocompromised	86 (3.8)
Cancer	67 (3.0)
**Stage at admission**	
Stage A (mild-to-moderate)	578 (25.6)
Stage B (severe)	1046 (46.3)
Stage C (critical)	633 (28.1)

**Table 2:** Therapeutic intervention and oxygen supplementation therapy.

Interventions or Oxygen Supplementation Therapy	Number (%)
Therapeutic or medical intervention used during hospitalization *	
Hydroxychloroquine or chloroquine	22 (1.0)
Triple combination therapy	226 (10.0)
Favipiravir	1581 (70.1)
Dexamethasone	1824 (80.0)
Remdesivir	243 (10.8)
Tocilizumab	274 (12.1)
COVID-19 Convalescent Plasma transfusion	36 (1.6)
Oxygen-supplementation therapy *	2203 (97.6)
Nasal or face mask	1563 (69.3)
CPAP	166 (7.4)
High-flow face mask	1283 (56.9)
BiPAP	594 (26.3)
IMV	1298 (57.5)
ECMO	22 (1.0)

* Some patients received multiple interventions or different types of oxygen therapy; thus, numbers may not add up to 100%. Triple combination therapy consists of lopinavir/ritonavir, ribavirin, and interferon beta-1b. Abbreviations: CPAP: continuous positive airway pressure; BiPAP: bilevel positive airway pressure; IMV: invasive mechanical ventilation; ECMO: extracorporeal membrane oxygenation.

**Table 3:** The odds ratios (OR) and 95% confidence interval (95%CI) from the multivariable logistic regression for the association between patients’ poor outcomes, as a composite outcome, and patients’ characteristics, comorbid conditions, and stage and symptoms at the time of admission.

Variables	Patients without Poor Outcomes *n* (%)	Patients with Poor Outcomes *n* (%)	*p*-Value	OR (95%CI)
**Patients’ characteristics**				
Age: 41–64 years vs. ≤ 40 years	441 (36.7)	761 (63.3)	**<0.001**	**1.82 (1.31, 2.52)**
65–75 years vs. ≤ 40 years	88 (19.0)	374 (81.0)		**4.79 (3.19, 7.18)**
≥ 75 years vs. ≤ 40 years	76 (20.3)	299 (79.7)		**4.04 (2.64, 6.17)**
Gender: Female vs. male	198 (31.1)	438 (68.9)	0.345	0.90 (0.72, 1.12)
Nationality: Non-Saudi vs. Saudi	407 (29.2)	987 (70.8)	0.469	0.92 (0.75, 1.15)
**Risk factors**				
Diabetes mellitus	429 (27.8)	1012 (70.2)	0.257	0.88 (0.70, 1.10)
Hypertension	363 (27.7)	945 (72.3)	0.886	0.98 (0.78, 1.24)
History of cardiovascular disease	124 (23.8)	397 (76.2)	0.282	1.16 (0.89, 1.52)
Obesity or severe obesity	105 (26.6)	290 (73.4)	**0.009**	**1.43 (1.10, 1.87)**
History of pulmonary disease	87 (25.4)	256 (74.6)	0.584	1.09 (0.81, 1.45)
Underlying end organ dysfunction	54 (19.2)	227 (80.8)	0.051	1.42 (1.00, 2.03)
Immunocompromised	19 (22.1)	67 (77.9)	0.443	1.28 (0.68, 2.39)
Cancer	18 (26.9)	49 (73.1)	0.599	0.84 (0.43, 1.63)
**Stage at admission:** Stage B vs. Stage A	379 (36.2)	667 (63.8)	**<0.001**	**6.46 (2.34, 17.8)**
Stage C vs. Stage A	73 (11.5)	560 (88.5)		**22.3 (11.0, 45.3)**
**Symptoms at the time of admission**				
High temperature	203 (43.6)	263 (56.4)	0.880	1.04 (0.66, 1.64)
Continuous cough	212 (42.8)	283 (57.2)	0.166	1.45 (0.86, 2.44)
Shortness of breath	226 (43.0)	299 (57.0)	**0.049**	**1.80 (1.00, 3.22)**
Loss of the sense of smell	51 (45.9)	60 (54.1)	0.057	0.62 (0.39, 1.01)
Fatigue and having muscle aches or headache	118 (38.1)	192 (61.9)	**<0.001**	**2.16 (1.47, 3.17)**
Respiratory rate ≥ 30/min	276 (33.4)	549 (66.6)	**0.037**	**1.42 (1.02, 1.96)**
Blood oxygen saturation ≤ 93%	373 (36.3)	653 (63.7)	0.064	0.46 (0.20, 1.05)
PaO_2_/FiO_2_ ratio < 300	106 (27.2)	283 (72.8)	**0.002**	**1.65 (1.21, 2.26)**
Lung infiltrates within 48 h of admission	242 (35.2)	445 (64.8)	0.910	1.02 (0.76, 1.36)
Sepsis	42 (14.3)	252 (85.7)	**0.002**	**0.41 (0.24, 0.71)**
Altered consciousness	25 (8.4)	272 (91.6)	**0.004**	**2.29 (1.30, 4.01)**

Numbers in bold represent significant results at α < 0.05.

**Table 4:** Patients’ composite outcome stratified by gender and stage at admission.

Variables	Patients without Poor Outcomes *n* (%)	Patients with Poor Outcome *n* (%)	*p*-Value	OR (95%CI)
**Gender**			0.748	
Male	516 (31.8)	1105 (68.2)		-
Female	198 (31.1)	438 (68.9)		-
**Stage at admission**			**<0.001**	
Stage A	262 (45.3)	316 (54.7)		**1**
Stage B	379 (36.2)	667 (63.8)		**1.46 (1.19, 1.79)**
Stage C	73 (11.5)	560 (88.5)		**6.36 (4.74, 8.53)**

Numbers in bold represent significant results at α < 0.05.

## Data Availability

The datasets used and/or analyzed during the current study are not publicly available due the presence of patients’ specific information and the limitation enforced by the IRB office in the MoH.

## Supplementary Materials

The following supporting information can be downloaded at: https://www.mdpi.com/article/10.3390/jcm11102818/s1, Figure S1: The ROC curve for the multivariable logistic regression model for the composite outcome.
