# Supplementary material for: Predictors of Poor Outcome among Critically Ill COVID-19 Patients: A Nationally Representative Sample of the Saudi Arabian Population
Source: J Clin Med. 2022 May 17;11(10):2818. doi: 10.3390/jcm11102818 (PMC9147701; doi:10.3390/jcm11102818)
Supplement: Supplementary file 1 [file jcm-11-02818-s001.zip › jcm-1688000-supplementary.pdf]

The model specifications

ROC curve and value

The ROC value for the multivariable logistic regression model for predicting the composite poor outcome was significantly better than no variables in the model 0.7472 with a p-value of ($p < 0.0001$) and the curve is provided in Figure S1.

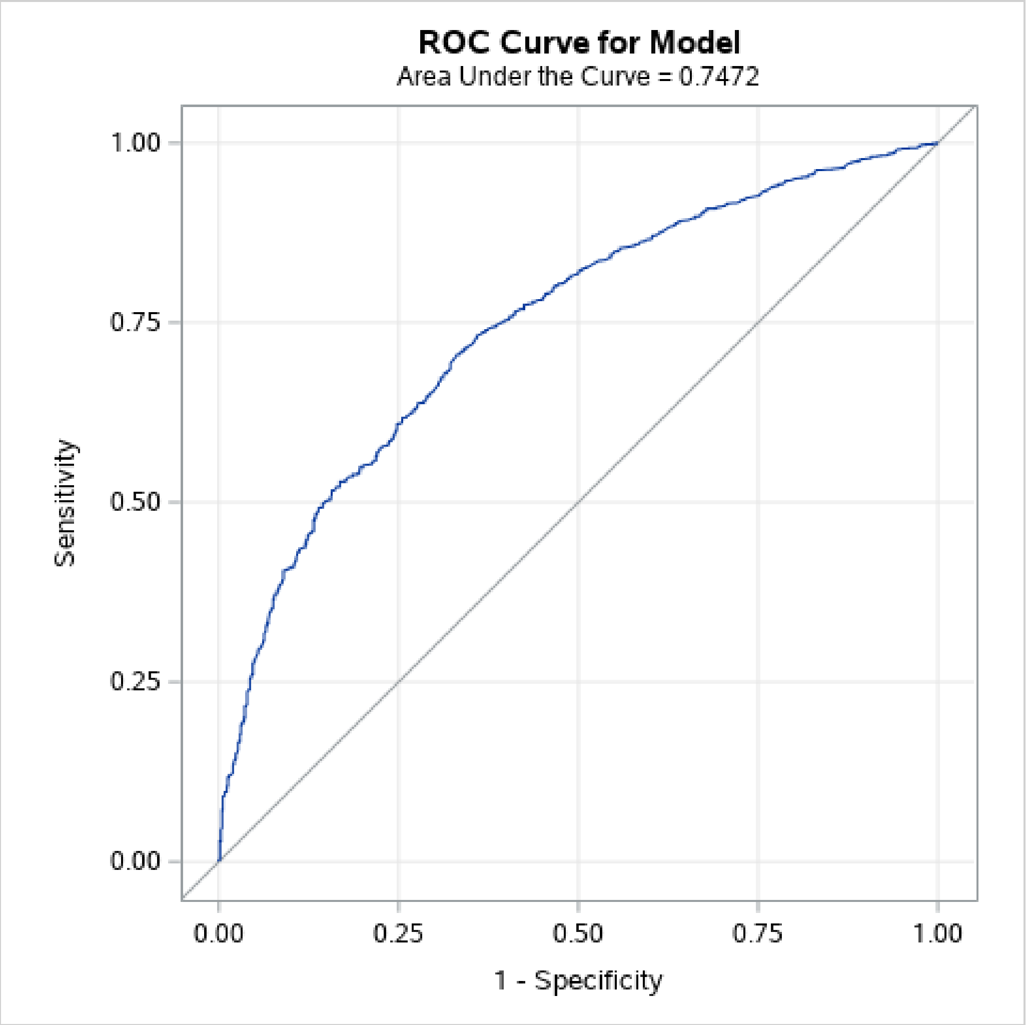

Figure S1. The ROC curve for the multivariable logistic regression model for the composite outcome.

Sensitivity, specificity, positive predictive value, and negative predictive value

At the optimal classification threshold of 0.64, based on the maximized Concordance Probability (CP) and Youden' Index, the sensitivity of the model was 70.7% with a specificity of 64.0%. The positive predictive value (PPV) at this threshold was 0.81 and the negative predictive value (NPV) was 0.50.
